# Two mixed-NH_3_/amine platinum (II) anticancer complexes featuring a dichloroacetate moiety in the leaving group

**DOI:** 10.1038/srep02464

**Published:** 2013-08-19

**Authors:** Weiping Liu, Jia Su, Jing Jiang, Xingyao Li, Qingsong Ye, Hongyu Zhou, Jialin Chen, Yan Li

**Affiliations:** 1 State Key Laboratory for Platinum Group Metals, Kunming Institute of Precious Metals, 650106 Yunnan, China; 2 State Key Laboratory of Phytochemistry and Plant Resources in West China, Kunming Institute of Botany, Chinese Academy of Sciences, 650201 Yunnan, China; 3 University of the Chinese Academy of Sciences, Beijing 100049, China

## Abstract

Two mixed-NH_3_/amine platinum (II) complexes of 3-dichoroacetoxylcyclobutane-1, 1-dicarboxylate have been prepared in the present study and characterized by elemental analysis and IR, HPLC-MS and ^1^H, ^13^C-NMR. The complexes exist in equilibrium between two position isomeric forms and undergo hydrolysis reaction in aqueous solution, releasing the platinum pharmacophores and dichloroacetate which is a small-molecular cell apoptosis inducer. Both complexes were evaluated for in vitro cytotoxic profile in A549, SGC-7901 and SK-OV-3 caner cells as well as in BEAS-2B normal cells. They exhibit markedly cytoxicity toward cancer cells by selectively inducing the apoptosis of cancer cells, whereas leaving normal cells less affected. They have also the ability to overcome the resistance of SK-OV-3 cancer cells to cisplatin. Our findings offer an alternative novel way to develop platinum drugs which can both overcome the drug resistance and selectively target tumor cells.

Platinum-based drugs represented by cisplatin, carboplatin and oxaliplatin have become fundamental components of standard chemotherapy regiments, and are widely used in antitumor therapy of testicular/ovarian tumors and lung/colorectal carcinomas^1^,^2^. Despite the therapeutic benefits, the efficacy of platinum-based treatment regiments is considerably compromised not only by severe side-effects but also by insurmountable cross-resistance^3^. Therefore efforts to circumvent platinum resistance continue to play an important role in the development of novel platinum drugs. Although a number of mechanisms are involved in drug resistance, failure to achieve cell death after the formation of platinum-DNA adduct might be an important factor^3^,^4^. One effective way of combating the resistance, as illustrated by recent studies^5^,^6^,^7^,^8^,^9^, is to combine the platinum pharmacophores with small-molecular cell apoptosis inducers to form dual-functional compounds so as to enhance the drug sensitivity and decrease the resistance via a synergistic effect between the two active components.

Dichloroacetate (DCA) is a small molecule which has been used for years to treat patients with mitochondrial diseases^10^. It was shown by recent reports^11^,^12^,^13^ to be able to trigger apoptosis through selectively targeting the mitochondria of cancer cells resistant to the anticancer drugs. Furthermore, unlike other anticancer drugs, DCA does not appear to affect normal cells^14^. This unique action of DCA gives a clue on the development of novel platinum drugs which are able to overcome the acquired resistance to cisplatin in certain type of cancer and avoid toxicity towards the normal cells at the meanwhile. The first platinum complex containing DCA moiety is mitaplatin reported in 2009 by Dhar and Lippard who demonstrated, mitaplatin, as a Pt (IV) compound with two DCA moieties directly coordinated to platinum in the axial positions, displayed a dual-killing mode and was considerably effective in cisplatin-resistant cells^9^. More recently, Dr Haihua described another Pt (IV) complex bearing an axial DCA ligand which also showed enhanced cytotoxicity^15^.

Inspired by this innovative research, we designed and synthesized a series of platinum (II) complexes featuring a dichloroacetate moiety tethered to the leaving group via ester bond (Fig. 1, 2). We expected that a synergistic anticancer action would be realized by combining the effects of platinum pharmacophore with DCA produced by hydrolysis in cancer cells. Among these complexes, we found that two mixed-NH_3_/amine (amine = cyclopentylamine, cyclohexylamine) platinum (II) complexes exhibited potent anticancer properties. In this paper, we report synthesis and cytotoxic profiles of the two complexes (**1a**, **2a**).

## Results

### Synthesis and characterization

**Figure 3:**
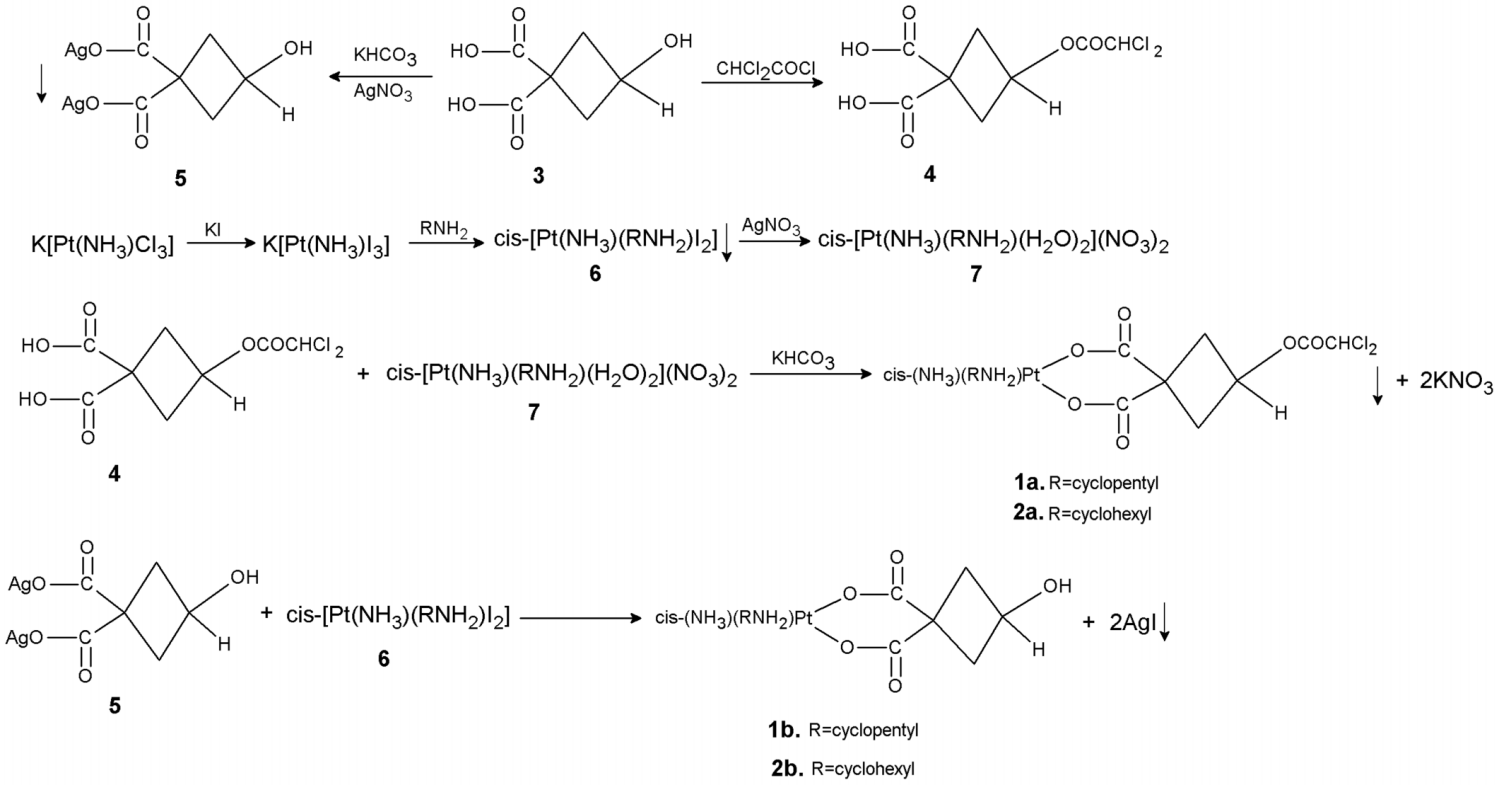
Synthetic routes of target complexes (1a, 2a) and their corresponding hydrolysis products (1b, 2b).

**Figure 4:**
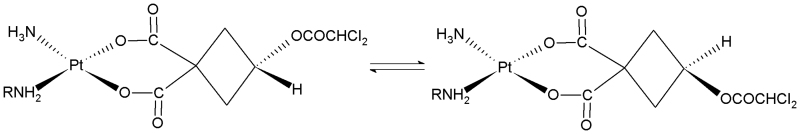
Equilibrium between two isomeric forms of target complexes (1a, 2a).

Cis-[Pt(NH_3_)(RNH_2_)I_2_], as an important intermiediate for mixed-NH_3_/amine platinum(II) complexes, was prepared according to a well-known method^16^. The target complexes **1a** and **2a** were synthesized following the routes shown in Fig. 3 with a yield of 50–55% based on the final reaction between **4** and **7**. Similar to cycloplatam^17^, both complexes existed in equilibrium between two isomeric forms due to 3-dichloroacetoxyl on the cyclobutane ring in either the cis or trans orientations in relation to NH_3 _(Fig. 4). The position isomeric phenomenon was confirmed by HPLC-Mass measurements in our studies. Under proper chromatographic conditions, two peaks developed at a different retention time, in addition to the peaks of the solvent, in the chromatograms (see Supplementary Fig. S1 and S2). They had nearly the same shape and area, and displayed exactly the same pattern of UV-visible absorption spectra and MS-ESI^+^ spectra with M^+^ at m/e 567 for **1a** and 581 for **2a**, the calculated molecular weights. The complexes also showed characteristic cluster ions in their mass spectra due to the isotopic peaks of platinum and chloride ion. Platinum has five total isomers out of which three have major abundance with 33% for ^194^Pt, 34% for ^195^Pt and 25% for ^196^Pt, whereas there are two ^35^Cl (76%) and ^37^Cl (24%) isotopes for chlorine. For a complex containing one platinum atom and two chlorine atoms^18^, the calculated main value distribution for the molecular ion cluster is 72:73:100:47:58. The most prominent ion observed in the mass spectra of both **1a** and **2a** was the [M + H]^+^ cluster. The isotopic peak distribution was 8.5% at m/z 565:8.6% at m/z 566:12% at m/z 567:5.5% at m/z 568:8.7% at m/z 569 for **1a**, and was 21% at m/z 579:23% at m/z 580:31% at m/z 581:24% at m/z 582:22% at m/z 583 for **2a**, roughly in agreement with the calculated values. We tried to separate the two isomeric forms but failed, for the complexes do not have a sufficient solubility in water/methanol solvent.

The chemical structures were characterized by elemental analysis, IR, ESI^+^-mass and ^1^H, ^13^C NNR. All the data and spectra (see Supplementary Fig. S3–S10) agreed well with the proposed structures. The introduction of COCHCl_2 _moiety to complexes **1a** and **2a** was evidenced by the appearance of C = O stretch band at 1748 cm^−1^ in the infrared spectra and C signals at 164 ppm in ^13^C NNR spectra. It is important to note that most of the carbons had two signals because of two isomeric forms co-existing in the complexes.

Both complexes were nearly insoluble in water or ethanol (≈0.1 mg/ml) and slightly soluble in methanol and DMSO, but soluble in polyethyleneglycol 400 which was chosen as the solvent to make the stock solutions of the complexes for the following biological testing. The hydrolysis rate of the complexes in 1:10 (v/v) solution of water and methanol determined by HPLC was less than 2% within 4 hours and the hydrolysis products were, as expected, dichloroacetate and **1b** for complex **1a**, or **2b** for complex **2a**. Complexes **1b** and **2b** were also synthesized for comparison in HPLC measurements as well as in biological tests. As **1b** and **2b** were found very soluble in water (>25 mg/ml) compared with **1a** or **2a**, another synthetic reaction was employed to produce the desired complexes. Accordingly complexes **1b** and **2b** also have two positional isomeric forms. All the complexes used in our studies were, in fact, a mixture of two isomeric forms.

### Cytotoxic profile

#### Selective cytotoxicity of the target complexes 1a and 2a on cancer cells

The cytotoxicity of **1a** and **2a** was determined by MTS assay along with DCA, carboplatin, cisplatin, **1b** and **2b** (hydrolysis products of **1a** and **2a**) as well as a mixture of **1b** and DCA against three human cancer cell lines representing three tumor entities: non-small cell lung carcinoma (A549), gastric carcinoma (SGC-7901) and ovarian carcinoma (SK-OV-3), and the normal lung epithelial cell line (BEAS-2B) as well. As shown in Table 1, DCA alone did not show any significant anticancer activity with the IC_50_ exceeding 200 μM, however the target complexes **1a** and **2a** bearing a DCA group exhibited obvious cytotoxicity against A549, SGC-7901 and SK-OV-3 cancer cells (IC_50_, 11.54–21.88 μM), only slightly less than cisplatin, but much greater than carboplatin and the corresponding hydrolysis products **1b** and **2b**. Noteworthy, **1a** was also much more active than the 1:1 molar mixture of **1b** and DCA, implying that a synergistic anticancer action had achieved between **1b** and DCA released from the hydrolysis of **1a**. Moreover, by comparing the IC_50_ values of the tested compounds towards cancer cell lines with those towards the normal lung epithelial cells BEAS-2B, **1a** and **2a** showed selective cytotoxicity on the cancer cells, whereas cisplatin, carboplatin and other compounds exhibited general cytotoxicity towards all the cells tested.

#### Selective apoptosis induced by 1a in A549 cancer cells over non-cancerous cells

**Figure 5:**
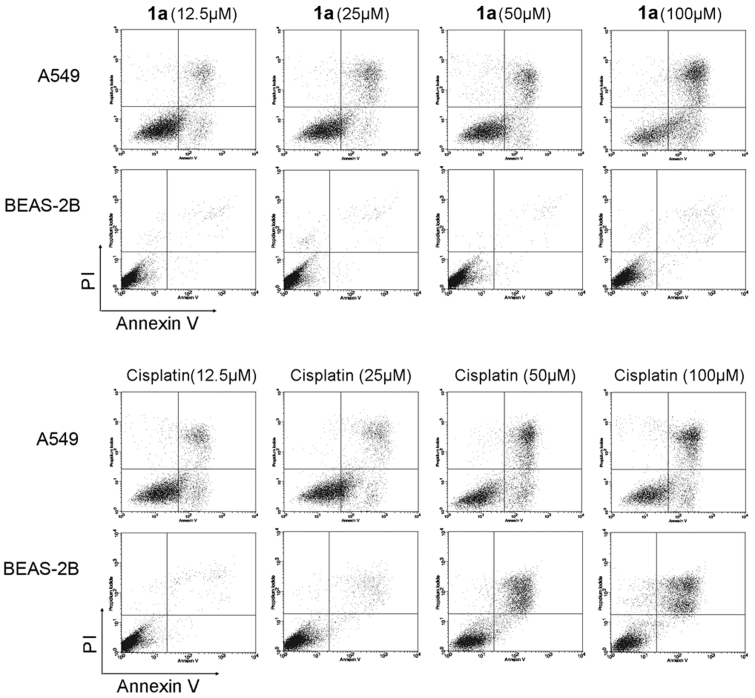
Complex 1a selectively induced cancer cell apoptosis. Cells were treated with **1a** or cisplatin for 24 h at indicated concentrations, and subjected to apoptosis analysis. Experiments were repeated three times and diagram of one representative experiment was shown.

Cell apoptosis was analyzed by Annexin-V-FITC/Propidium iodide staining and flow cytometry. Apoptosis induced and the quantified ratios of apoptotic cells under the treatment of **1a** or cisplatin at indicated dosages are shown in Fig. 5 and Table 2 respectively. Similar to cisplatin, **1a** significantly induced apoptosis of A549 cells in a dose-dependent manner with a apoptosis ratio of 18.93% at the concentration of 12.5 μM. Surprisingly, it had little effect on normal human lung bronchial epithelial cells even at the concentration of 100 μM (apotoic ratio, 2.88), indicating that **1a** may have a tumor cell-selective apoptosis-inducing property. On the contrary, obvious apoptosis induced by cisplatin was observed in both A549 cancer cells and the BEAS-2B normal human lung bronchial epithelial cells when the concentration is over 25 μM.

#### Cytotoxicity of the complexes 1a and 2a in cisplatin-resistant SK-OV-3 cell line

Cross-resistance profile of the complexes **1a** and **2a** was evaluated. Resistance Index was defined as the ratio of IC_50_ value of resistant cells to that of sensitive cells. SK-OV-3 and cisplatin-resistant SK-OV-3 cells (SK-OV-3/DDP) cell lines were chosen for MTS assay because drug resistance frequently occurred in the chemotherapy of ovarian tumors. As expected, **1a** and **2a** displayed similar cytotoxic activity against both sensitive and cisplatin-resistant human ovarian cancer cells with the resistance index being nearly equal to 1, indicating that they have the potential to overcome the resistance of cancer cells to cisplatin (Table 3). The results may be attributed to the synergistic effect between the platinum pharmacophores and DCA released by the hydrolysis of **1a and 2a** in the cells.

## Discussion

Development of new platinum anti-cancer drugs remains an important field in medicinal chemistry and attracts extensive interests from both academic institutions and pharmaceutical industry. In the past ten years much effort has been devoted to non-classical platinum complexes but without success. No any new platinum complex has been approved for clinical use since the year of 2001. Therefore, direct structural modification of classical platinum drugs by introducing bioactive groups could still be an effective way to develop new-generation drugs.

In the present study, two mixed-NH_3_/amine platinum (II) complexes of 3-dichoroacetoxylcyclobutane-1,1-dicarboxylate have been prepared, characterized and evaluated for their cytotoxicity profile. Both complexes exist in equilibrium between two isomeric forms due to 3-dichloroacetoxyl on the cyclobutane ring in either the *cis* or *trans* orientations in relation to NH_3_. They undergo hydrolysis in water, releasing two active species, the platinum pharmacophores and dichloroacetate. The two complexes exhibit markedly cytotoxicity in cancer cells by selectively inducing apoptosis of cancer cells, and exert little effect on normal human cells. They have the potential to overcome the resistance of cancer cells to cisplatin, probably due to the synergistic effect between the platinum pharmacophores and dichloroacetate. These two complexes**,** as neutral and relatively lipophilic molecules, are also expected to enter the cells more easily and bring more dichloroacetate into cells than free dichloroacetate alone which exists as anion in physiological.</url> PH condition. These results offer an alternative novel way to develop platinum drugs which can both overcome the drug resistance and target tumor cells selectively.

## Methods

### Chemistry

#### General

K[Pt(NH_3_)Cl_3_], a commercially available platinum compound, was purchased from Alfa Aesar and 3-hydroxy-1,1-cyclobutanedicarboxylic acid 3 was prepared as previously described^19^,^20^Composition analyses for C, H and N were performed with a Carlo-Ebra instrument, whereas the content of platinum was determined according to the method in EP6.5. LC/MS measurements were carried out on a Waters Acquity Xevo TQ-S in ESI^+^ mode using MeOH/H_2_O as the solvent. FT-IR spectra were measured in KBr pellets with a Perkin Elmer 880 spectrometer. ^1^H, ^13^C NMR spectra were recorded in DMSO on Brucker DRX-500 MHz relative to TMS (tetramethylsilane) as an external standard. A VG Autospec was also used to measure FAB^+^ spectra with glycerol (Gly) as the matrix.

#### Chromatographic conditions

An Agilent Zorbax SB-C18 column (4.6 × 250 mm, 5 μm) was used with CH_3_OH/H_2_O as the mobile phase. The flow rate was 1.0 ml/min, and the column temperature is 40°C. The detection wavelength was 238 nm and the injection volume was 10 μl.

#### Preparation of 3-dichoroacetoxylcyclobutane-1,1-dicarboxylic acid **4**

3-hydroxycyclo- butane-1,1-dicarboxylic acid (10 g, 62.5 mmol) was dissolved in 100 ml acetone, and subsequently 10 ml (105 mmol) CHCl_2_COCl was added. The mixture was stirred at 45°C for 4 hours. The solvent was removed under reduced pressure and a yellow dish residue was obtained. The residue was dissolved by isopropyl ether and the solution was concentrated to produce a white solid product which was then purified by re-crystallization from isopropyl ether. Yield: 6.6 g (39%). m.p.148–150°C. IR (KBr, cm^−1^): 3012–2900 (w, *ν*_C-H_), 1762 (s, *ν*_C = O_), 1713 (s, *ν_as_*_(COOH)_). ^13^C NMR (500 MHz, DMSO): δ 36.2 (*C*H_2_), 46.3 (*C*(COOH)_2_), 64.8 (*C-*O-COCHCl_2_), 66.9 (*C*HCl_2_), 163.7 (*C* = O),171.6, 171.6 (2*C*OOH), analysis (calcd., found for C_8_H_8_Cl_2_O_6_): C (35.4, 35.1), H (2.95, 3.01).

#### Preparation of cis-[Pt (NH_3_)(RNH_2_) I_2_] **6**

K[Pt(NH_3_)Cl_3_] (10 g, 28 mmol) was dissolved in water (100 ml) and treated with KI ( 20.9 g, 126 mmol). After standing for 40 min at room temperature, a solution of cyclopentylamine or cyclohexylamine (28 mmol in 50 ml water) was added dropwise. The mixture was stirred for 4 h and the yellow precipitate obtained was filtrated off, washed with water and ethanol and dried in vacuo at 55°C. Yield: 88% (13.6 g) for cis-[Pt (NH_3_) (C_5_H_11_NH_2_)I_2_], 91% (14.4 g) for cis-[Pt(NH_3_)(C_6_H_13_NH_2_)I_2_]. For cis-[Pt (NH_3_)(C_5_H_11_NH_2_)I_2_], analysis (calcd, found): Pt (35.4, 35.1). For cis-[Pt (NH_3_) (C_6_H_13_NH_2_) I_2_], analysis (calcd, found): Pt (34.5, 34.8).

#### Preparation of cis-[Pt(NH_3_)(RNH_2_)(3-dichoroacetoxylcyclobutane-1,1-dicarboxylate)] **1a, 2a**

To a suspension of cis-[Pt(NH_3_)(RNH_2_)I_2_] (6.00 mmol) in 40 ml distilled water, 2.039 g (12.00 mol) AgNO_3_ in 10 ml distilled water was added, and the mixture was stirred for 24 hours in the dark at 35°C. After AgI formed was filtrated off, the filtrate was mixed with a freshly prepared aqueous solution of dipotassium 3-dichoroacetoxylcyclobutane-1, 1-dicarboxylate to produce a white precipitate. It was collected by filtration, washed with distilled water and ethanol, dried under vacuum at 35°C. Yield: 51% (1.73 g) for complex **1a**, 47% (1.64 g) for complex **2a**.

### Complex 1a

Found (% calculated for C_13_H_20_Cl_2_N_2_O_6_Pt): Pt 34.1 (34.5), C 27.8 (27.6), H 3.55 (3.53), N 4.92 (4.95). MS-ESI^+^ m/z: 589 ([M + Na]^+^,23%), 567([M + H]^+^,13%). IR (KBr, cm^−1^): 3213, 3124 (m, *v*_N-H_), 2958, 2872 (m, *v*_C-H_), 1748 (vs, *v*_C = O_), 1618 (vs, *v*_as(COO)_), 1352 (vs, *v*_a(COO)_). IR (KBr, cm^−1^): 3213, 3124 (m, *v*_N-H_), 2958, 2872 (m, *v*_C-H_), 1748 (vs, *v*_C_ = O), 1618 (vs, *v*_as(COO)_), 1352 (vs, *v*_a(COO)_). ^1^H NMR (500 MHz, DMSO): δ 1.48 (≈4H, m, 2C*H_2_*, cyclopentyl-C3), 1.64, 1.95 (≈4H, m, 2C*H_2_*, cyclopentyl-C2), 2.30, 3.05 (≈4H, m, 2C*H_2_*, cyclobutyl-C2 ), 3.10 (≈1H, m, C*H*, cyclopentyl-C1), 3.82 (≈1H, m, C*H*, cyclobutyl-C3), 4.12 (≈3H, m, N*H*_3_), 4.92 (≈1H, m, OCOC*H*Cl_2_), 5.01 (≈2H, m, N*H*_2_). ^13^C NMR (500 MHz, DMSO): δ 23.4, 23.5, 23.6, 23.7 (2 × 2*C*-3, cyclopentyl), 30.1, 30.7 32.7, 33.1(2 × 2*C*-2, cyclopentyl), 42.0, 42.1 (2 × 2*C*-2, cyclobutyl), 48.1, 51.2 (2*C*-1, cyclobutyl), 56.8, 56.9(2*C*-1, cyclopentyl), 60.3, 60.7 (2*C*-3, cyclobutyl), 67.1, 70.4 (2*C*HCl_2_), 164.8(2C = O), 177.2,177.3, 177.5, 177.6 (2 × 2*C*OO^−^). MS-ESI^+^ (m/z): 589 (M + Na^+^, 23%), 567(M + H^+^, 13%). Anaysis (calcd., found for C_13_H_20_Cl_2_N_2_O_6_Pt): Pt (34.5, 34.1), C (27.6, 27.8), H (3.53, 3.55), N (4.95, 4.92).

### Complex 2a

Found (% calculated for C_14_H_22_Cl_2_N_2_O_6_Pt)): Pt 33.6 (33.8), C 28.8 (29.0), H 3.81 (3.79), N 4.85 (4.83). MS-ESI^+^ m/z: 603 ([M + Na]^+^,50%), 581([M + H]^+^,30%). IR (KBr, cm^−1^): 3219, 3124 (m, *v*_N-H_), 2934, 2856 (m, *v*_C-H_), 1749 (vs, *v*_C = O_), 1620 (vs, *v*_as(COO)_), 1354 (vs, *v*_a(COO)_). ^1^H NMR (500 MHz, DMSO): δ 1.04, 1.53 (≈2H, m, C*H_2_*, cyclohexyl-C4), 1.48 (≈4H, m, 2C*H_2_*, cyclohexyl-C3), 1.68, 2.24 (≈4H, m, 2C*H_2_*, cyclohexyl-C2) 2.36, 3.07 (≈4H, m, 2C*H_2_*, cyclobutyl-C2 ), 2.68 (≈1H, m, C*H*, cyclopentyl-C1), 3.82 (≈1H, m, C*H*, cyclobutyl-C3), 4.11 (≈3H, m, N*H*_3_), 4.93 (≈1H, m, OCOC*H*Cl_2_), 4.99 (≈2H, m, N*H*_2_). ^13^C NMR (500 MHz, DMSO): δ 24.4, 24.5 (2*C*-4, cyclohexyl), 24.6, 24.8, 24.9, 25.2 (2 × 2*C*-3, cyclohexyl), 30.3, 31.9 32.1, 33.2(2 × 2*C*-2, cyclohexyl), 41.5, 41.9, 42.0, 42.1 (2 × 2*C*-2, cyclobutyl), 48.0, 49.2 (2*C*-1, cyclobutyl), 54.4, 54.5(2*C*-1, cyclohexyl), 60.2 60.7 (2*C*-3, cyclobutyl), 67.1, 70.4 (2*C*HCl_2_), 164.9(2C = O), 177.2,177.3, 177.5, 177.6 (2 × 2*C*OO^−^). MS-ESI^+^ (m/z): 603 (M + Na^+^,50%), 581(M + H^+^,30%). Anaysis (calcd., found for C_14_H_22_Cl_2_N_2_O_6_Pt): Pt (33.8, 33.6), C (29.0, 28.8 ), H (3.79, 3.81), N (4.83, 4.85).

#### Preparation of cis-[Pt(NH_3_)(RNH_2_)(3-hydroxy-cyclobutane-1,1-dicarboxylate)] **1b, 2b**

cis-[Pt(NH_3_)(RNH_2_)I_2_] (6.00 mmol) suspended in 100 ml distilled water was mixed with 2.243 g, 6.00 mmol disilver 3-hydroxy-cyclobutane-1,1-dicarboxylate. The mixture was stirred for 48 hours in the dark at 35°C. After AgI formed was filtrated off, the filtrate was condensed at 45°C under reduced pressure to 5 mL, a white crystalline product precipitated and then it was filtrated off, washed with cool distilled water and ethanol, and dried in a vacuum oven at 45°C. Yield: 53% (1.45 g) for complex 1b, 56% (1.58 g) for complex 2b.

### Complex 1b

IR (KBr, cm^−1^): 3215, 3122 (m, *v*_N-H_), 2956, 2870 (m, *v*_C-H_), 1637 (vs, *v*_as(COO)_), 1376 (vs, *v*_a(COO)_). ^13^C NMR (500 MHz, DMSO): δ 23.6(*C*-3, cyclopentyl), 32.7(*C*-2, cyclopentyl), 42.1 (*C*-2, cyclobutyl), 48.1 (*C*-1, cyclobutyl), 56.9(*C*-1, cyclopentyl), 60.3 (*C*-3, cyclobutyl), 177.2,177.6 (2*C*OO^−^). MS-FAB^+^ (m/z): 548 (M^+^ + Gly, 10%), 456 (M^+^, 100%). Analysis (calcd., found for C_11_H_20_ N_2_O_5_Pt): Pt (42.9, 42.5), C (29.0, 28.8), H (4.39, 4.43), N (6.15, 6.11).

### Complex 2b

IR (KBr, cm^−1^): 3219, 3125 (m, *v*_N-H_), 2932, 2855 (m, *v*_C-H_), 1639 (vs, *v*_as(COO)_), 1376 (vs, *v*_a(COO)_). ^13^C NMR (500 MHz, DMSO): δ: 24.4(*C*-4, cyclohexyl), 25.2(*C*-3, cyclohexyl), 33.2(*C*-2, cyclohexyl), 42.0 (*C*-2, cyclobutyl), 48.1 (*C*-1, cyclobutyl), 54.4(*C*-1, cyclohexyl), 60.2 (*C*-3, cyclobutyl), 177.2,177.6 (2*C*OO^−^). MS-FAB^+^ (m/z): 470 (M^+^, 13%), 98(cyclohexylamine^+^, 100%). Analysis (calcd., found for C_12_H_22_ N_2_O_5_Pt): Pt (41.6, 42.5), C (30.7, 28.8), H(4.69, 4.29), N (5.97, 5.54).

### Biology

#### Cell culture

Human lung cancer (A549), human normal lung epithelial cell line (BEAS-2B), human stomach cancer (SGC-7901) and human ovarian cancer (SK-OV-3) were purchased from ATCC, whereas cisplatin-resistant SK-OV-3 cells (SK-OV-3/DDP) were kindly provided by Chinese Academy of Medical Sciences (Beijing, China). Cells were grown in DMEM or RPMI-1640 medium (Hyclone, USA) containing 10% fetal bovine serum. Both media were supplemented with 100 units/ml of penicillin and 100 μg/ml of streptomycin. Cells were maintained at 37°C in a humidified incubator with an atmosphere of 5% CO_2_.

#### MTS assay

Cytotoxicity was determined by performing MTS assay. Briefly, 100 μl of cells suspension were seeded in 96-well cell culture plates and allowed to adhere overnight. The cells were treated with drugs for 48 hours, and then 20 μl of CellTiter 96® AQ_ueous_ One Solution Reagent (Promega, Madison, USA) was added and the cells were further incubated at 37°C for 1–2 h. Cell viability was measured by reading the absorbance at a wavelength of 490 nm. Concentrations of 50% inhibition of growth (IC_50_) were calculated on the basis of the relative survival curve.

#### Cell apoptosis assay

To analyze the cells for apoptosis, cells were plated and allowed to adhere overnight. Cells were treated with drugs indicated for 24 hours and then analyzed for apoptosis using Annexin-V-FITC/Propidium iodide staining. Cells were trypsinized, pelleted, washed in PBS, and resuspended in 1×binding buffer containing Annexin-V-FITC and propidium iodide (BD Pharmingen) according to the manufacturer's instructions. The samples were analyzed for the apoptosis using a FACSCalibur flow cytometer (BD Biosciences, Franklin Lakes, NJ).

## Author Contributions

W.L., J.J., Q.Y. and J.C. conducted the experiments of the chemistry. J.S., X.L. and H.Z. conducted the experiments of biology. Y.L. and W.L. designed experiments, analyzed and interpreted the data, and wrote the manuscript.

## Supplementary Material

Supplementary Information Two mixed-NH3/amine platinum (II) anticancer complexes featuring a dichloroacetate moiety in the leaving group

## Figures and Tables

**Figure 1:**
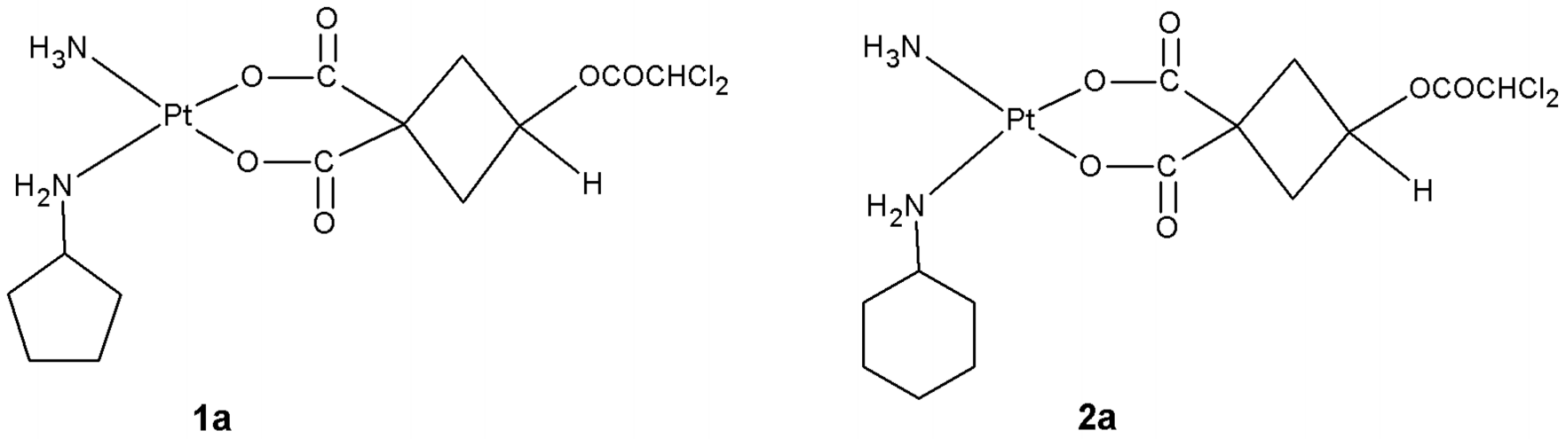
Chemical structures of designed complexes.

**Figure 2:**
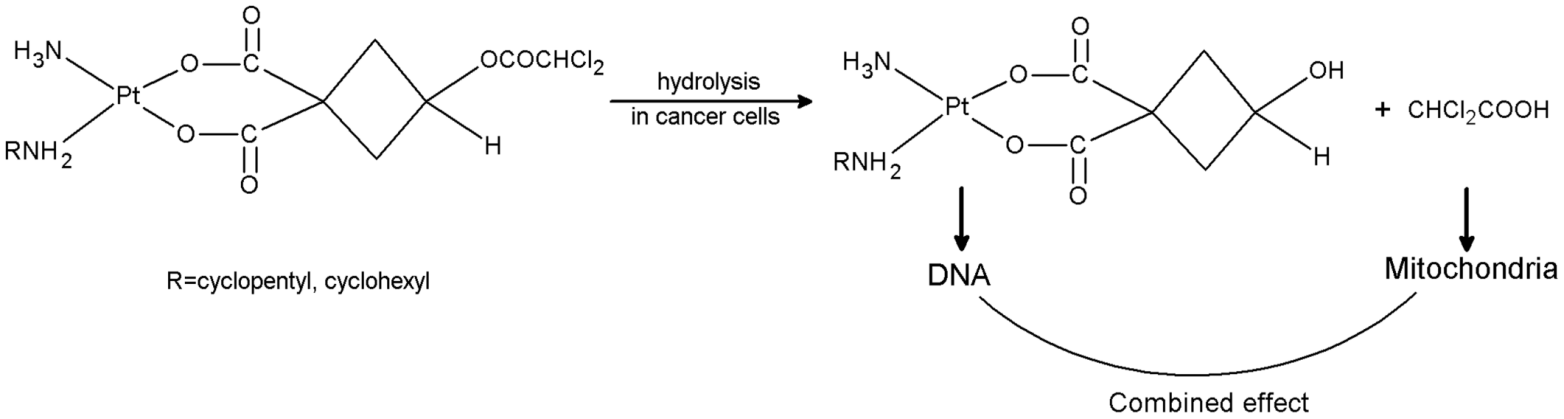
Expected synergistic mechanism of designed complexes.

**Table 1:** Cytotoxicity of tested compounds (t = 48 h, n = 3)

	IC_50 _(mean ± SD, μM)			
Treatment	A549	SGC-7901	SK-OV-3	BEAS-2B
DCA	>200	>200	>200	>200
Carboplatin	141.39 ± 11.30	127.1 ± 11.82	65.35 ± 3.55	74.44 ± 2.51
Cisplatin	14.77 ± 1.02	13.56 ± 1.03	5.04 ± 0.25	11.67 ± 0.51
**1a**	17.09 ± 1.55	21.88 ± 1.96	18.05 ± 0.85	61.45 ± 1.50
**2a**	15.34 ± 1.12	16.69 ± 0.82	11.54 ± 0.91	52.09 ± 3.12
**1b**	61.47 ± 7.98	56.22 ± 4.88	74.25 ± 2.64	79.94 ± 2.57
**2b**	71.53 ± 5.21	69.53 ± 2.31	78.20 ± 1.85	75.03 ± 4.83
**1b** + DCA (1:1 mole)	65.23 ± 3.18	56.27 ± 5.53	82.10 ± 1.53	63.15 ± 2.97

**Table 2:** Quantification of cell apoptosis induced by 1a and cisplatin (t = 24 h, n = 3)

		Apoptosis, % (mean ± SD)	
Treatment	Concentration (μM)	A549	BEAS-2B
**1a**	12.5	18.93 ± 3.83	0.71 ± 0.45
	25	26.76 ± 2.33	1.24 ± 1.73
	50	40.93 ± 9.66	1.27 ± 0.92
	100	67.46 ± 8.06	2.88 ± 2.15
Cisplatin	12.5	20.09 ± 3.06	2.64 ± 1.43
	25	32.84 ± 5.48	5.86 ± 1.30
	50	50.51 ± 6.42	38.93 ± 5.26
	100	56.28 ± 6.91	42.80 ± 3.40

**Table 3:** Cytotoxicity of 1a and 2a in cisplatin-resistant SK-OV-3 cell line (t = 48 h, n = 3)

	IC_50_ (mean ± SD, μM)		
Treatment	SK-OV-3	SK-OV-3/DDP	Resistance Index
Cisplatin	5.15 ± 1.38	19.15 ± 0.94	≈4
**1a**	17.8 ± 3.97	18.05 ± 2.41	≈1
**2a**	10.09 ± 2.38	11.09 ± 0.63	≈1
