# Supplementary material for: Two mixed-NH3/amine platinum (II) anticancer complexes featuring a dichloroacetate moiety in the leaving group
Source: Sci Rep. 2013 Aug 19;3:2464. doi: 10.1038/srep02464 (PMC3746205; doi:10.1038/srep02464)
Supplement: Supplementary Information — Two mixed-NH3/amine platinum (II) anticancer complexes featuring a dichloroacetate moiety in the leaving group [file srep02464-s1.pdf]

Two mixed-NH_3/amine platinum (II) anticancer complexes featuring a dichloroacetate moiety in the leaving group

Weiping Liu, Jia Su, Jing Jiang, Xingyao Li, Qingsong Ye, Hongyu Zhou, Jialin Chen, Yan Li*

Supplementary Information

Figure S1. HPLC chromatogram (the mobile phase: CH_3OH/H_2O (V/V) =30/70) of complex **1a** and the corresponding UV and MS-ESI$^+$ spectra of the two isomeric forms.

Figure S2. HPLC chromatogram (the mobile phase: CH_3OH/H_2O (V/V)=27/73) of complex **2a** and the corresponding UV and MS-ESI$^+$ spectra of the two isomeric forms.

Figure S3. IR spectra of 3-dichoroacetoxylcyclobutane-1,1-dicarboxylic acid **4**.

Figure S4. IR spectra of complex **1a.**

Figure S5. IR spectra of complex **2a.**

Figure S6. ^{13}C NMR spectra of 3-dichoroacetoxylcyclobutane-1,1-dicarboxylic acid.

Figure S7A. ^{13}C NMR spectra of complex **1a** in DMSO.

Figure S7B. ^{13}C NMR spectra of complex **1a** in DMSO with white background.

Figure S8A. ^{13}C NMR spectra of complex **2a** in DMSO.

Figure S8B. ^{13}C NMR spectra of complex **2a** in DMSO with white background.

Figure S9A. ^{1}H NMR spectra of complex **1a** in DMSO.

Figure S9B. ^{1}H NMR spectra of complex **1a** in DMSO with white background.

Figure S10A. ^{1}H NMR spectra of complex **2a** in DMSO.

Figure S10B. ^{1}H NMR spectra of complex **2a** in DMSO with white background.

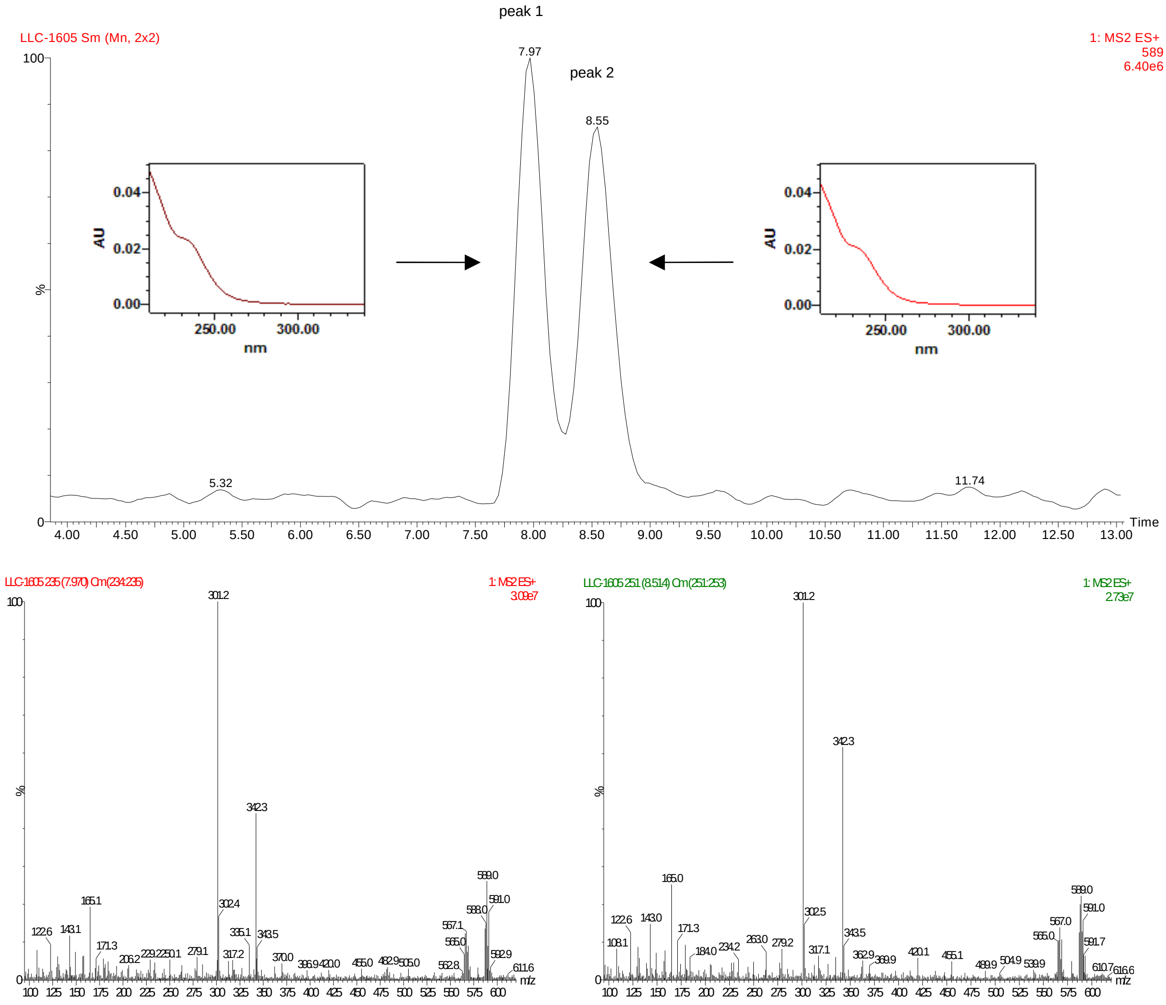

peak 1: retention time=7.97min

peak 2: retention time=8.55min

Figure S1. The HPLC chromatogram (the mobile phase: CH_3OH/H_2O (V/V) =30/70) of complex **1a** and the corresponding UV and MS-ESI$^+$ spectra of the two isomeric forms.

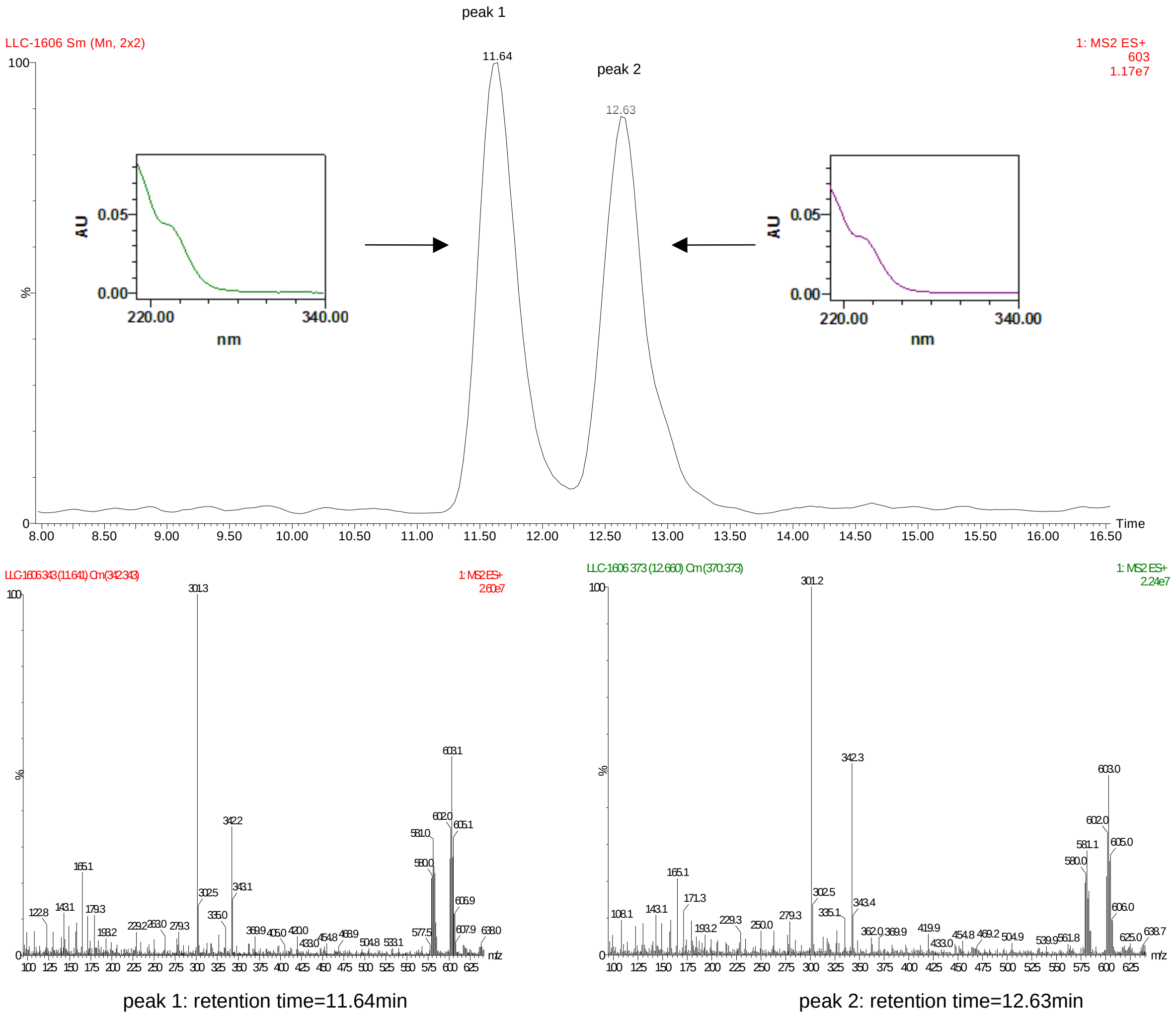

Figure S2 The HPLC chromatogram (the mobile phase: CH_3OH/H_2O (V/V)=27/73) of complex **2a** and the corresponding UV and MS-ESI$^+$ spectra of the two isomeric forms.

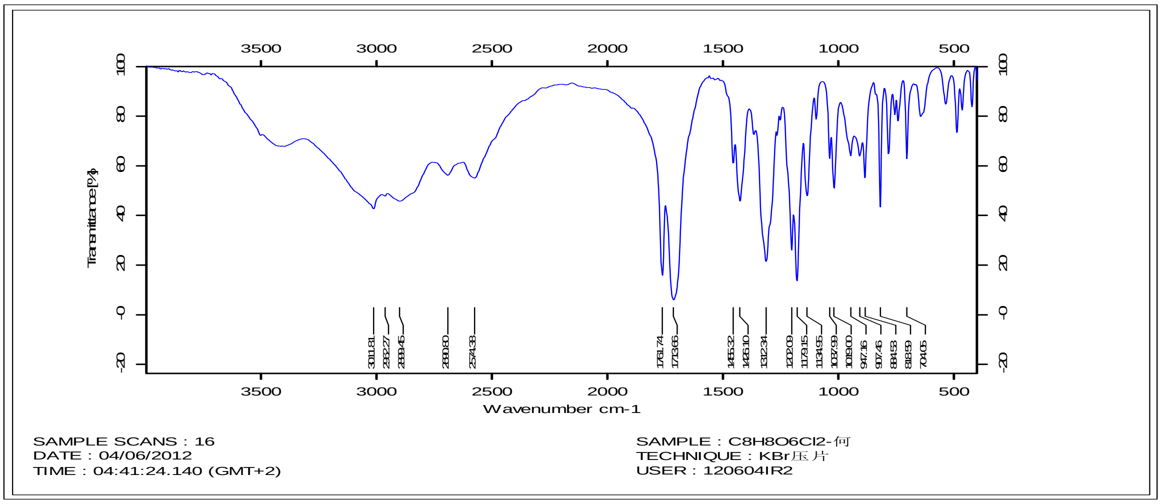

Figure S3. IR spectra of 3-dichoroacetoxylcyclobutane-1,1-dicarboxylic acid **4**.

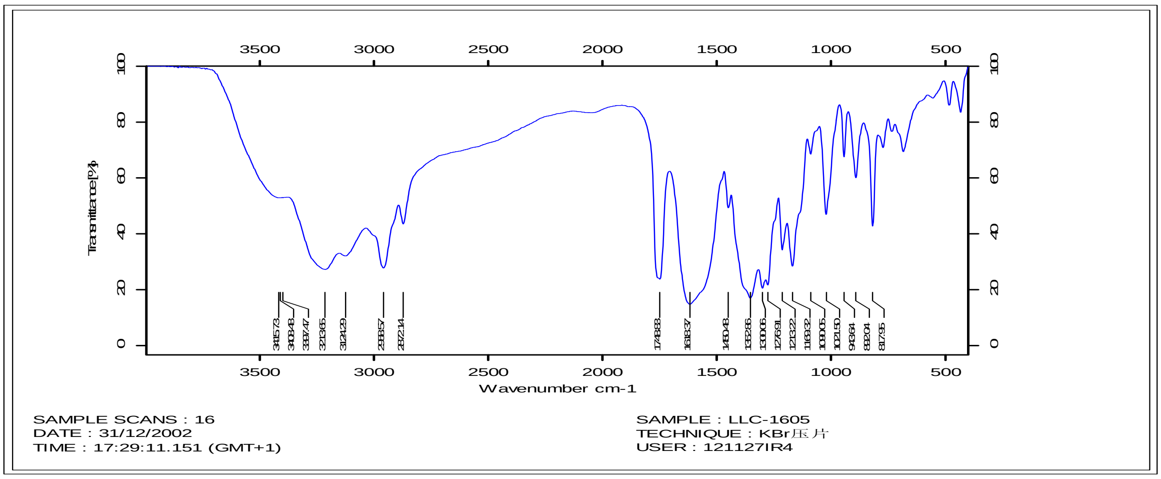

Figure S4. IR spectra of complex **1a.**

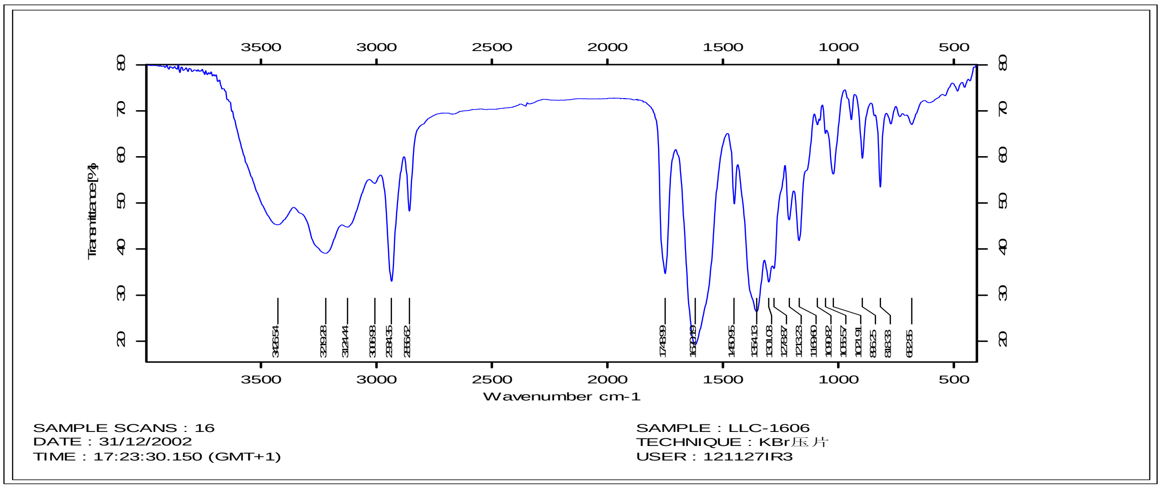

Figure S5. IR spectra of complex **2a.**

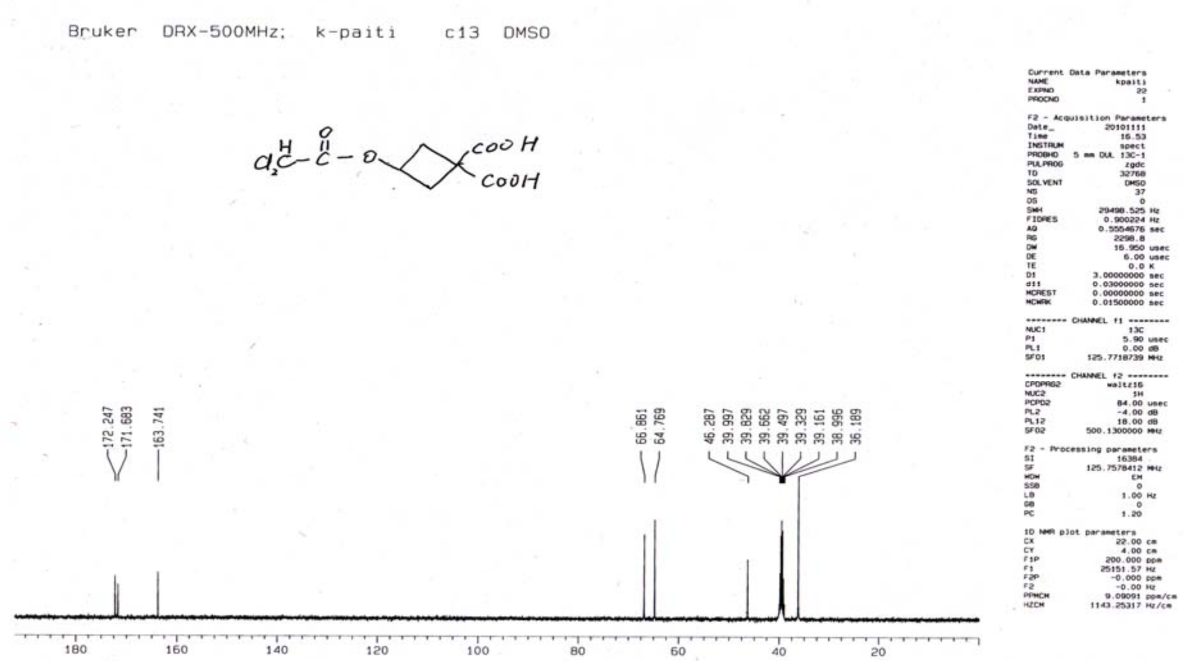

Figure S6. ^{13}C NMR spectra of 3-dichoroacetoxylcyclobutane-1,1-dicarboxylic acid.

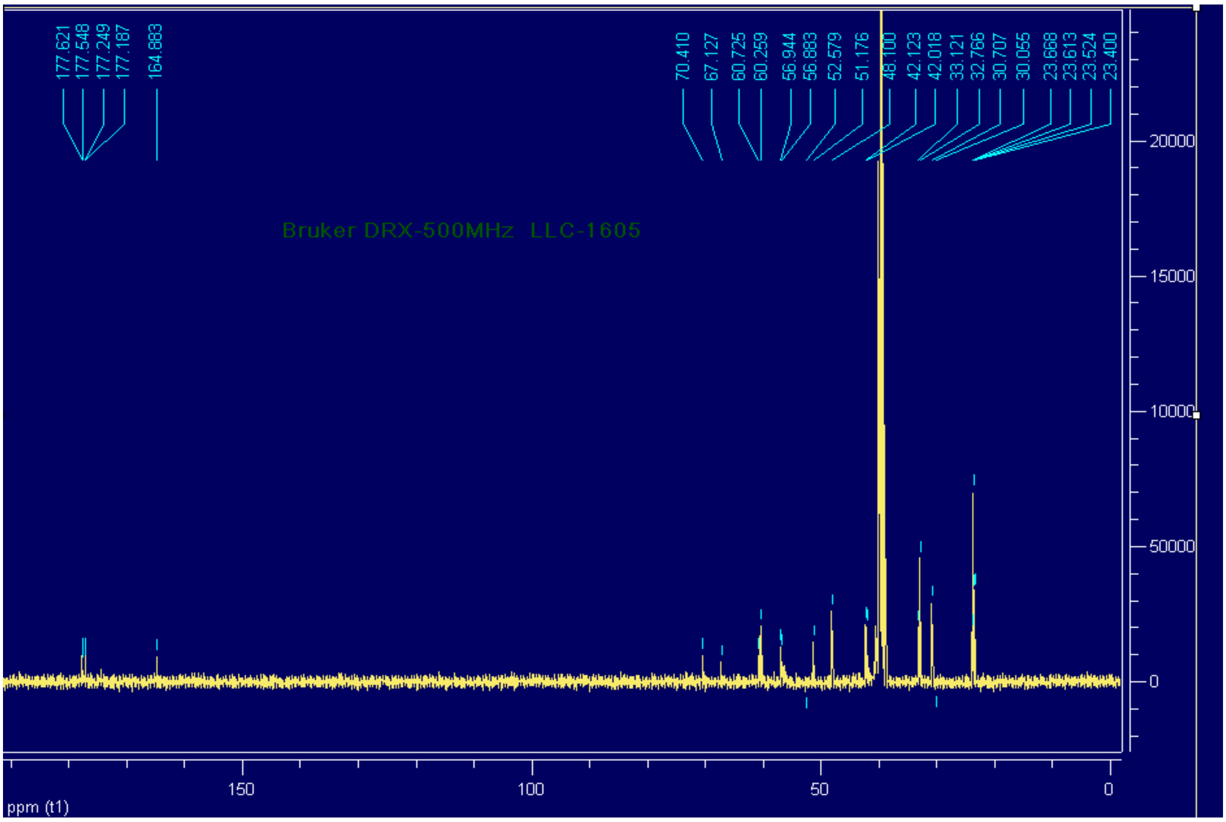

Figure S7A. ^{13}C NMR spectra of complex **1a** in DMSO.

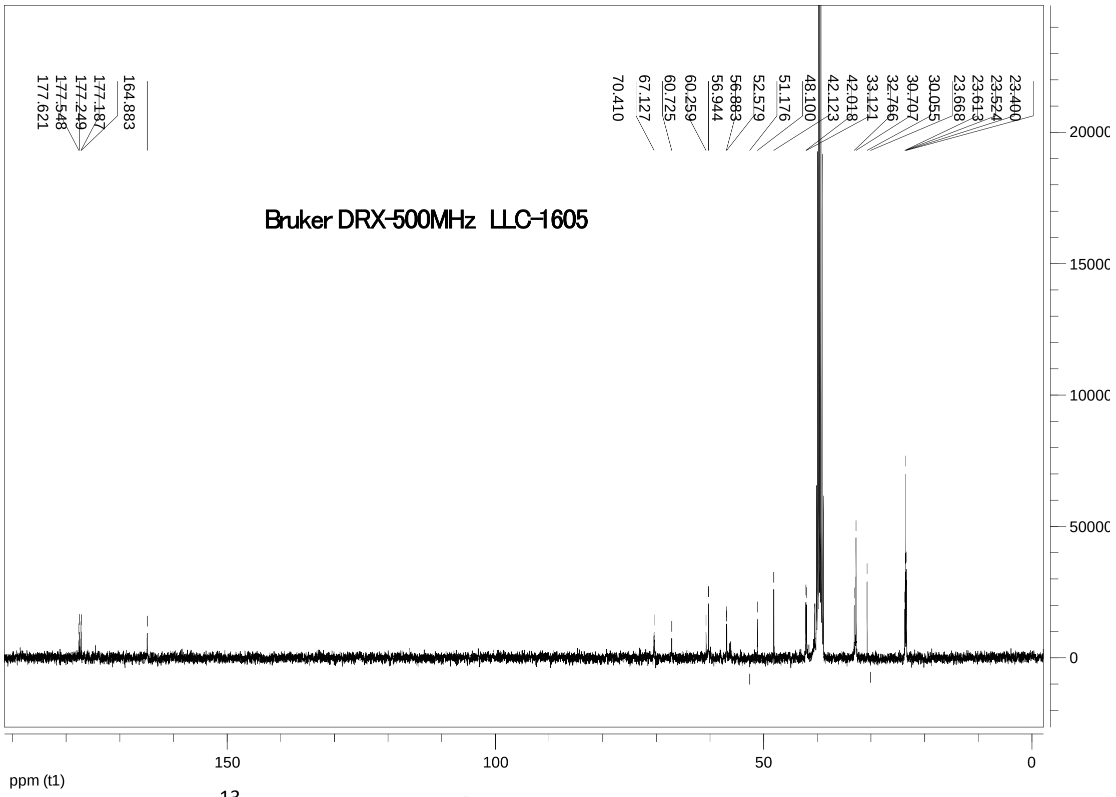

Figure S7B. ^{13}C NMR spectra of complex **1a** in DMSO with white background.

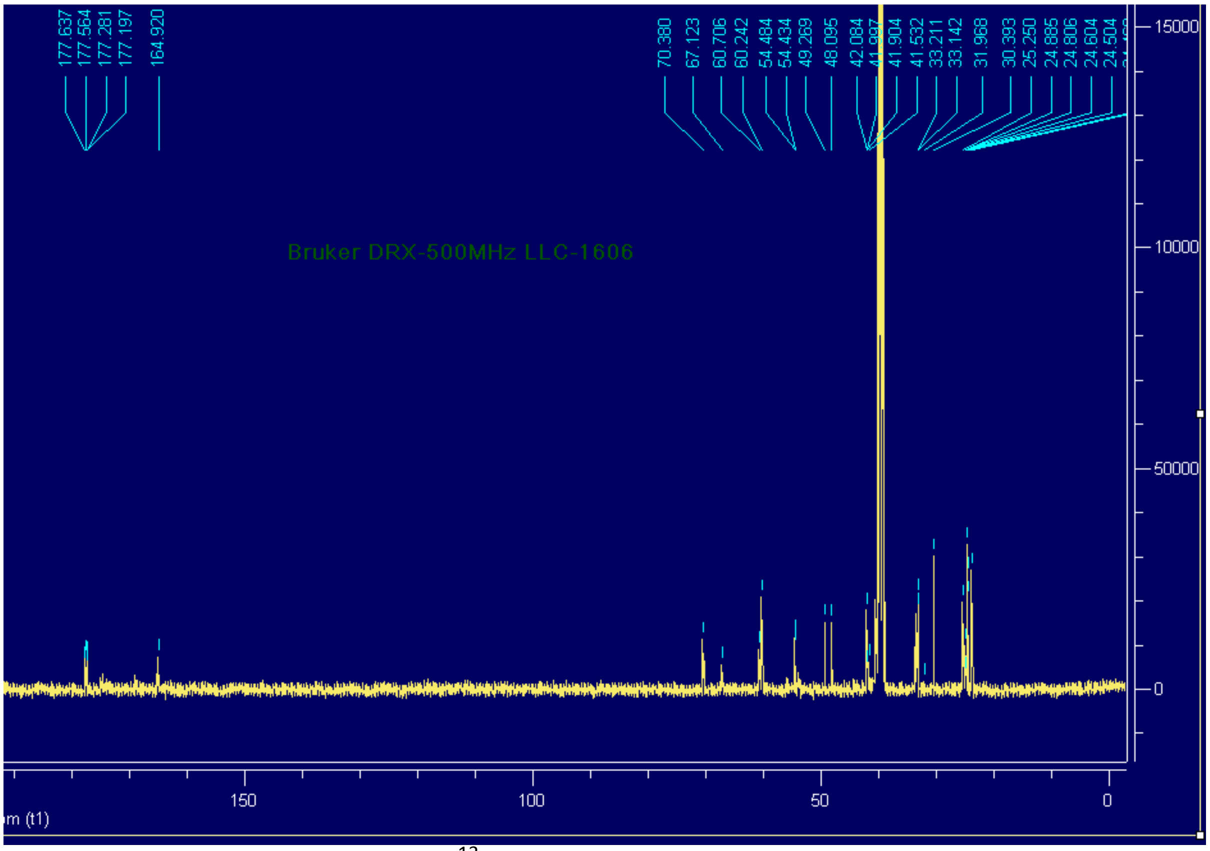

Figure S8A. ^{13}C NMR spectra of complex **2a** in DMSO.

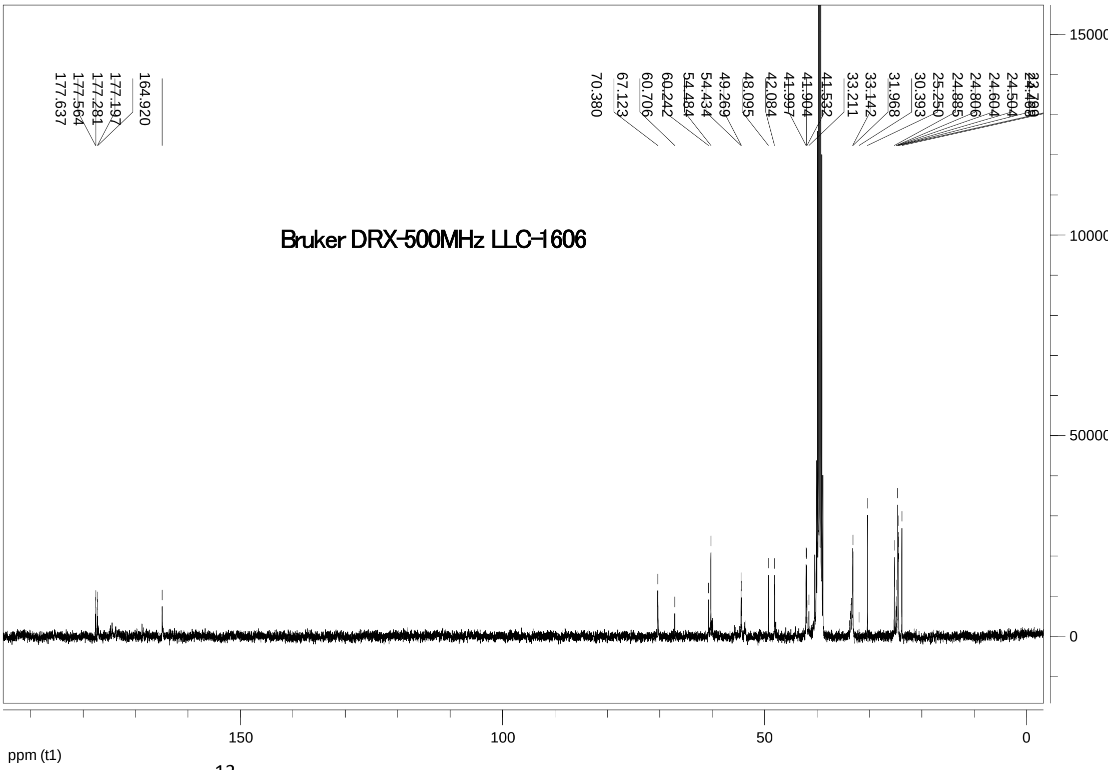

Figure S8B. ^{13}C NMR spectra of complex **2a** in DMSO with white background.

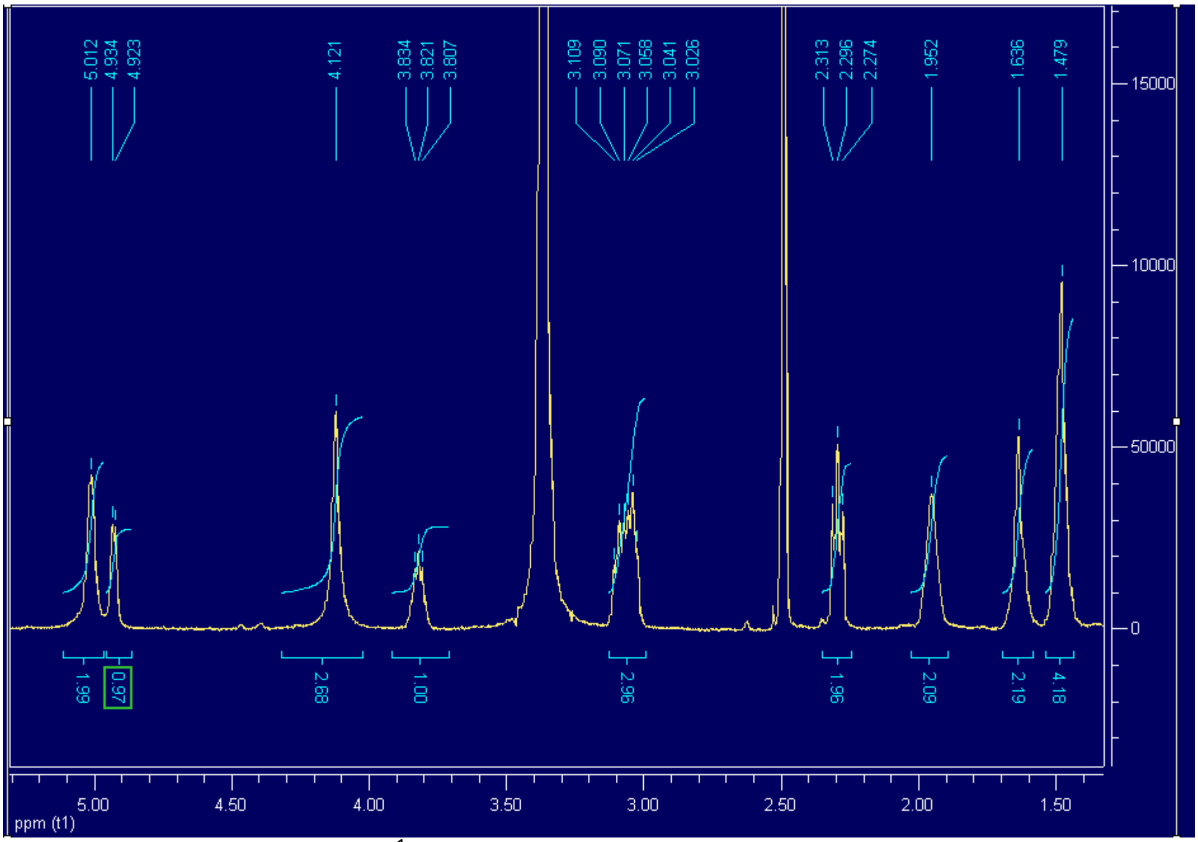

Figure S9A. ^{1}H NMR spectra of complex **1a** in DMSO.

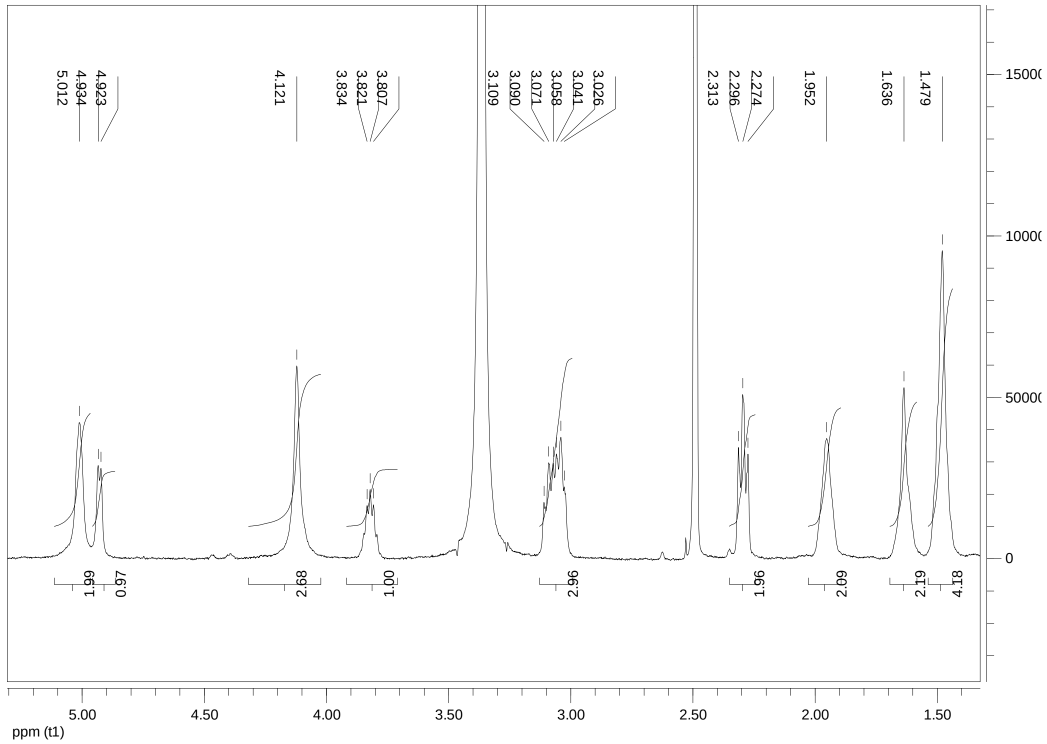

Figure S9B. ^{1}H NMR spectra of complex **1a** in DMSO with white background.

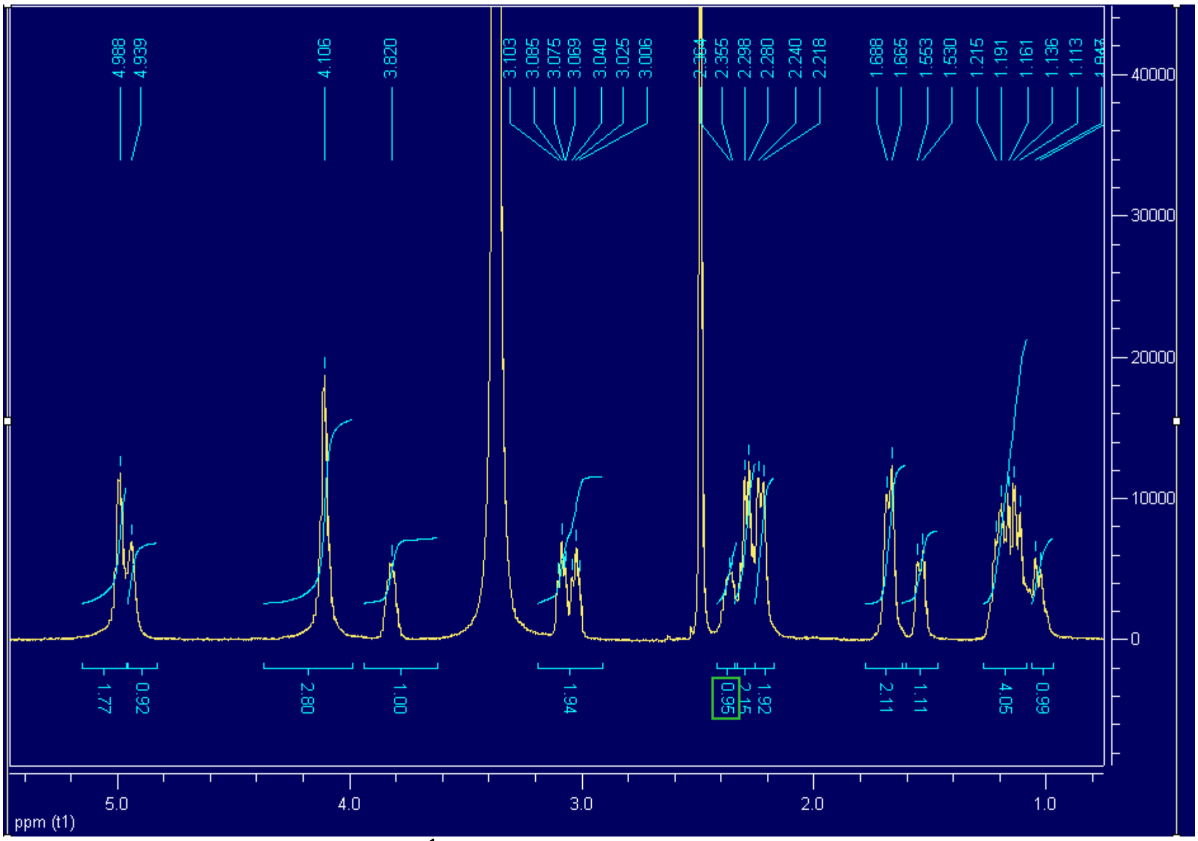

Figure S10A. ^{1}H NMR spectra of complex **2a** in DMSO.

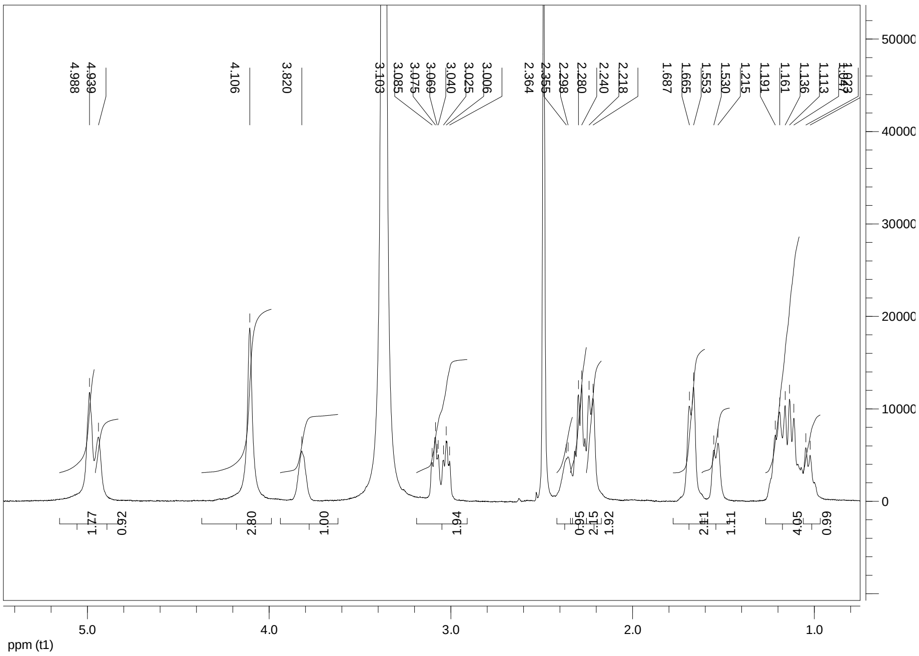

Figure S10B. ^{1}H NMR spectra of complex **2a** in DMSO with white background.
